# Simulated Galactic Cosmic Radiation Exposure-Induced Mammary Tumorigenesis in *Apc*^Min/+^ Mice Coincides with Activation of ERα-ERRα-SPP1 Signaling Axis

**DOI:** 10.3390/cancers16233954

**Published:** 2024-11-26

**Authors:** Kamendra Kumar, Jerry Angdisen, Jinwenrui Ma, Kamal Datta, Albert J. Fornace, Shubhankar Suman

**Affiliations:** 1 Department of Oncology, Lombardi Comprehensive Cancer Center, Georgetown University Medical Center, Washington, DC 20057, USA; 2 Department of Biochemistry and Molecular & Cellular Biology, Georgetown University Medical Center, Washington, DC 20057, USA

**Keywords:** space radiation, breast cancer, estrogen, estrogen receptor-α, estrogen-related receptor α, secreted phosphoprotein 1, osteopontin

## Abstract

Background: Exposure to galactic cosmic radiation (GCR) is a breast cancer risk factor for female astronauts on deep-space missions. However, the specific signaling mechanisms driving GCR-induced breast cancer have not yet been determined. Methods: This study aimed to investigate the role of the estrogen-induced ERα-ERRα-SPP1 signaling axis in relation to mammary tumorigenesis in female *Apc*^Min/+^ mice exposed to simulated GCR (GCRsim) at 100–110 days post-exposure. Results: In GCRsim-exposed mice, we observed marked elevations in serum estradiol, increased ductal overgrowth, ERα activation, and upregulation of ERα target genes with pro-tumorigenic functions in mammary tissues that was coupled with a higher mammary tumorigenesis, relative to control. Additionally, the ERα target gene *Esrra*, which encodes ERRα, was also upregulated along with its oncogenic target gene *Spp1*, indicating the activation of the ERα-ERRα-SPP1 axis in mouse mammary tissues after GCRsim exposure. Using a human tissue microarray and human breast cancer gene expression analysis, we also highlighted the conserved nature of the ERα-ERRα-SPP1 signaling in human breast cancer development. Conclusions: We identified the ERα-ERRα-SPP1 signaling axis as a potential key mediator in GCR-induced breast cancer with conserved activation in human breast cancer. These findings suggest that targeting this pathway could serve as a potential target for therapeutic intervention to safeguard female astronauts during and after a prolonged outer space mission.

## Simple Summary

Female astronauts on deep-space missions are at increased risk of breast cancer due to exposure to galactic cosmic radiation (GCR). This study aimed to understand how GCR might lead to breast cancer by focusing on a hormone-related pathway involving estrogen receptor alpha (ERα) and other related molecules (ERRα and SPP1). In mice exposed to simulated GCR, we observed increased levels of estrogen, changes in breast tissue growth, and activation of genes that promote tumor formation. Similar results were found when analyzing human breast cancer tissues, suggesting that this pathway is also important in human breast cancer. These findings highlight the ERα-ERRα-SPP1 pathway as a key player in radiation-induced breast cancer risk, providing a potential target for developing protective therapies to safeguard female astronauts during deep-space missions.

## 1. Introduction

Female astronauts are considered to be at an increased risk of developing breast cancer due to occupational exposure to ionizing radiation (IR) during space missions [1,2]. This risk is primarily inferred from epidemiological studies of women exposed to low-linear energy transfer (LET) IR, such as γ-rays and X-rays [3,4,5,6]. On Earth, the average radiation background is approximately 2.4 mSv per year, whereas astronauts aboard the International Space Station (ISS) are exposed to radiation doses of around 0.5 mSv per day [7,8]. In deep space, beyond Earth’s magnetosphere, the IR dose rate from galactic cosmic radiation (GCR) is estimated to be around 1 mSv per day, and for longer-duration missions, such as those to Mars, the estimated cumulative dose ranges from 0.30 to 0.45 Gy (equivalent to 0.87 to 1.20 Sv) [9]. Unlike low-LET IR on Earth, GCR consists of highly energetic protons, alpha particles, and heavy ions [10,11]. High-LET heavy ions, due to their ability to penetrate current spacecraft shielding, can induce persistent oxidative stress, genotoxic damage, accelerated senescence, and a senescence-associated secretory phenotype (SASP)-driven inflammatory response, thereby contributing to an elevated cancer risk [12,13,14,15,16].

Since no female astronaut has ventured into deep space, understanding the adverse health risks and associated molecular perturbations after space radiation exposure requires ground-based in vivo studies using simulated GCR (GCRsim) exposures [17]. The female *Apc*^Min/+^ mice harboring a germline mutation in one allele of the *Apc* (adenomatous polyposis coli) gene has been successfully used to study mammary tumorigenesis after IR exposure and is considered as a suitable surrogate model of human breast cancer, specifically for studying IR-induced mammary tumor initiation, progression, and associated molecular signaling events [18,19,20].

In women, activation of estrogen receptor-alpha (ERα), encoded by the *Esr1* gene, plays a crucial role in the initiation, development, and progression of breast cancer [21,22,23]. Similarly, studies using IR-exposed animal models have demonstrated a persistent estrogenic response (PER), characterized by elevated systemic estrogen levels and activation of ERα in mammary epithelial cells [19,24,25]. This response coincides with upregulation of oncogenic markers and an increased frequency of hormone receptor-positive tumors [19]. In addition to PER activation, female *Apc*^Min/+^ mice exposed to GCRsim have shown increased accumulation of secreted phosphoprotein 1 (SPP1, also known as osteopontin or OPN) in the ductal epithelium, which serves as a preneoplastic marker for breast cancer risk [20]. Notably, SPP1 expression in mammary epithelial cells has been implicated in mammary cancer initiation, and elevated plasma levels of SPP1 protein are associated with increased tumor burden [26,27,28]. However, the molecular links between IR-induced PER signaling and SPP1 are not yet well understood.

In addition to ERα, estrogen-related receptor alpha (ERRα), encoded by the *Esrra* gene, is another critical factor in both ERα positive and negative in breast cancers [29,30,31]. ERRα is frequently overexpressed in ER-positive human breast cancers and is associated with poor prognosis, contributing to increased tumor aggressiveness [32,33]. Due to the structural similarity in the DNA binding domain of ERRα and ERα, they are likely to compete and modulate the expression of a similar set of genes implicated in breast cancer development [34,35]. Furthermore, activation of both ERα and ERRα has been implicated in the overexpression of SPP1 [36,37,38,39,40]. Therefore, understanding GCRsim-induced alterations in the signaling axis involving ERα, ERRα, and SPP1 is crucial for elucidating the molecular events associated with GCRsim-induced mammary cancer risk.

In this ground-based mouse model study, we exposed female *Apc*^Min/+^ mice to GCRsim at NASA’s Space Radiation Laboratory (NSRL) and focused on understanding the roles of PER, ERRα, and SPP1 signaling in GCRsim-exposed *Apc*^Min/+^ mice to elucidate their contributions to breast cancer development. GCRsim exposure was associated with a PER characterized by elevated serum estradiol levels and increased activation of ERα, along with enhanced expression of downstream target genes in mammary tissues. Additionally, ERRα expression was elevated, and there was an accumulation of the preneoplasia marker SPP1 in GCRsim-exposed mice, indicating a synergistic effect of both receptors on mammary cancer progression. Notably, the overexpression of ERα, ERRα, and SPP1 was further corroborated by their presence in human breast cancer tissues, suggesting a conserved mechanism across species. This study aims to elucidate the molecular mechanisms underlying GCRsim-induced breast cancer risk, providing insights that could inform future pharmacological investigations to safeguard female astronauts during long-duration space missions

## 2. Materials and Methods

### 2.1. Mice and Radiation Exposure

**Figure 1:**
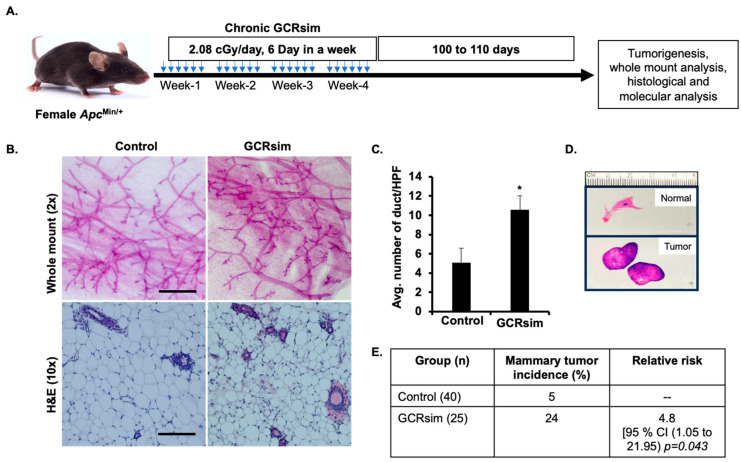
GCRsim exposure promotes mammary tissue overgrowth and increased duct density. (**A**) Schematic representation of the experimental setup depicting chronic exposure to GCRsim, using a 33-ion mixed beam to simulate the deep-space environment. Experimental animals were exposed to 2.08 cGy per day, 6 days per week, for 4 weeks, resulting in a cumulative dose of 50 cGy. (**B**) Representative images of mammary tissues: whole-mount micrographs (scale bar = 500 µm) show the extent of ductal branching, while H&E-stained sections (scale bar = 100 µm) highlight histological features. (**C**) Quantification of ductal density, expressed as the number of ducts per high-power microscopic field (HPF). (**D**) Representative H&E-stained normal mammary gland and tumor samples. (**E**) Comparison of mammary tumor incidence between control and GCRsim-exposed animals, showing increased tumor formation in the 50 cGy irradiated GCRsim group. Statistically significant difference (*p* < 0.05) relative to the control group is denoted by an asterisk (*).

Male *Apc*^Min/+^ mice in C57BL6 background were bred with wild-type female C57BL6 mice at the Georgetown University (GU) animal facility, and all female pups were genotyped to identify *Apc*^Min/+^ mice, as described on the Jackson Laboratory website (https://www.jax.org/Protocol?stockNumber=002020&protocolID=529, accessed on 1 October 2015). The female *Apc*^Min/+^ mice were randomly assigned to the respective experimental groups and were transported to the Brookhaven National Laboratory (BNL, Upton, NY, USA) animal facility using an approved laboratory animal courier service. Following one week of acclimatization, at the age of 8 to 10 weeks, mice were either sham irradiated or irradiated to 50 cGy of chronic GCRsim beam (Figure 1A). The 4-week irradiation period (at 2.08 cGy/day, 6 days a week, delivering a total dose of 50 cGy) in mice corresponds to 2–3 years of human age, aligning with the estimated duration of a manned Mars mission. This irradiation protocol effectively models the chronic, low-dose exposure astronauts would experience over an extended period. Although condensed into 4 weeks, the dose rate and cumulative dose are biologically relevant, providing an experimental timeline that simulates the long-term, low-dose radiation conditions of space while ensuring feasibility for animal studies. To closely mimic an “actual-GCR”-like scenario and dose rates, we irradiated animals to the full-spectrum GCRsim developed by NSRL at BNL that consists of seven different ion species (^1^H, ^2^He, ^6^C, ^16^O, ^28^Si, ^44^Ti, and ^26^Fe) and a wide energy spectrum (20–1000 MeV/n) [10,11,41]. Both at GU and BNL facilities, all animals were group-housed (5 per cage) and maintained with standard laboratory conditions including specific pathogen-free (SPF) environment and 12 h:12 h shift of light–dark cycles with easy access to water and food. All experiments were conducted in accordance with the Institutional Animal Care and Use Committee (IACUC) approved protocol # 2019-0070 at GU and #515 at BNL.

### 2.2. Biospecimen Collection, Tumor Counting, and Histological Assessments

The mice were euthanized using carbon dioxide (CO_2_) asphyxiation at 100 to 110 days post-irradiation and surgically dissected to reveal the mammary fat pads, and the macroscopic mammary tumors were quantified. Other pairs of mammary tissues and serum were flash-frozen and stored at −80 °C for further studies. Samples of normal-appearing mammary tissues and tumors were preserved in 10% buffered formalin for 24 h and then transferred to 70% ethanol prior to paraffin embedding and sectioning. Hematoxylin and eosin (H&E) staining was performed on formalin-fixed, paraffin-embedded (FFPE) mouse mammary gland sections of 5 μm thickness. The sections were deparaffinized in xylene, rehydrated through a graded ethanol series, and stained with hematoxylin for nuclear visualization, followed by eosin for cytoplasmic staining. Finally, digital images of the H&E-stained tissue sections were obtained using a bright-field microscope (Olympus BX63, Olympus America Inc., Center Valley, PA, USA), and the number of ducts in a given microscopic field were visually quantified at 200X magnification.

### 2.3. Mammary Gland Whole Mount Staining

Freshly isolated normal appearing mammary glands (i.e., mammary fat pad without any visible tumor node under a dissecting scope) were carefully spread and mounted on a microscope slide, and whole-mount staining was performed using the VitroView™ Mammary Gland Whole Mount Stain Kit (VB-3001, VitroVivo Biotech, Rockville, MD, USA), according to the manufacturer’s instructions. Briefly, the mammary glands were fixed in Carnoy’s fixative for 2 h, followed by a wash in 70% ethanol. The tissues were then rehydrated through graded ethanol concentrations, including 50% and 30% ethanol, and subsequently rinsed in distilled water. Staining was performed using carmine alum staining solution, with tissues incubated overnight at room temperature. The next day, the tissues were subjected to a series of dehydration steps using graded ethanol concentrations (70%, 90%, and 100%), followed by clearing in xylene. Finally, the tissues were mounted using Permount mounting medium (SP15-100, Fisher Chemical, Frederick, MD, USA), and digital images were obtained using a bright-field microscope followed by visual assessments of ductal morphology in irradiated and non-irradiated mammary gland tissues.

### 2.4. Serum Immunoassays

SPP1 and estradiol levels in serum samples were determined using immunoassay kits following the manufacturer’s instructions. SPP1 concentrations in 50-fold diluted serum samples from both control and chronic GCRsim-irradiated mice were quantified by using RayBio^®^ Mouse Osteopontin (SPP1) ELISA Kit (ELM-OPN; RayBiotech, Peachtree Corners, GA, USA). The Mouse SPP1 ELISA had a minimum detectable concentration of 4 pg/mL. Estradiol levels were measured in 2-fold diluted serum samples using the RayBio^®^ Human/Mouse/Rat Estradiol EIA Kit (EIAM-E2; RayBiotech). The estradiol EIA standard curve range was 0.1–1000 ng/mL, with a minimum detectable concentration of 1.5 ng/mL.

### 2.5. Immunohistochemistry and Image Quantification

Protein expression of hormone receptors (ERα and ERRα) and down downstream targets (Cyclin D1) were analyzed using immunohistochemistry. Formalin-fixed paraffin-embedded (FFPE) mammary tissue sections were deparaffinized and rehydrated, followed by a thermal antigen retrieval step using either citrate buffer (pH 6.0) (SKU:64142-08; Electron Microscopy Sciences, Hatfield, PA, USA) or Tris EDTA buffer (pH 9.0) (10-0037; Torrance, CA, USA). Tissue sections were incubated overnight in a humidified chamber at 4 °C with primary antibodies, ERα (ab32063; dilution 1:200; Abcam, Boston, MA, USA), ERRα (ab137489; dilution 1:100; Abcam, Boston, MA, USA), SPP1 (SC-21742; Santa Cruz, Dallas, TX, USA) and Cyclin D1 (MA5-14512; dilution 1:100; Invitrogen, Waltham, MA, USA). Finally, immunohistochemical signals were detected using a Mouse and Rabbit Specific HRP/DAB IHC Detection Kit (ab236466, Abcam, Boston, MA, USA). Further, sections were counter-stained with hematoxylin (SKU: 26043-06; Electron Microscopy Sciences), dehydrated and mounted using Permount mounting medium (SP15-100, Fisher Chemical, Frederick, MD, USA). Digital images (10–12 per group) were acquired and saved in TIFF format using cellSens Entry v1.15 software (Olympus, Center Valley, PA, USA). Quantification of IHC stained sections was performed using Fiji (ImageJ2) software v2.9.0/1.53t, either by counting the number of positively stained nuclei per high power field (HPF) or by measuring the diaminobenzidine (DAB) chromogen signal intensity within the defined region of interest (ROI) [42].

### 2.6. mRNA Expression Analysis

Total RNA was isolated from the flash-frozen mammary tissues by using Qiagen RNeasy mini kit (74104; Qiagen, Germantown, MD, USA). RNA purity was measured by the A260 nm/A280 nm ratio using a NanoDrop spectrophotometer (ND-1000, Thermo Scientific, Waltham, MA, USA). Next, 2 μg of RNA was reverse-transcribed into cDNA using iScript cDNA Synthesis Kit (1708891; Bio-rad, Hercules, CA, USA), according to the manufacturer’s instructions. Finally, quantitative real-time RT-PCR (qPCR) was performed to assess mRNA expression of *Esr1*, *Esrra*, *Ccnd1*, *cMyc*, and *Nrip1* genes using SsoAdvanced Universal SYBR Green Supermix (Cat # 1725271, Bio-Rad, Hercules, CA, USA) on a real-time PCR system (CFX96; Bio-rad, Hercules, CA, USA) as per the manufacturer’s instructions. PrimeTime qPCR premixed primer assays for all genes (Supplementary Table S1) were obtained from Integrated DNA Technology (www.idtdna.com, accessed on 21 September 2022). All predesigned primeTime qPCR primer sets are reported to achieve >90% efficiency. Amplification specificity for each PCR reaction was verified using a melting curve analysis and mRNA expression was analyzed by the comparative Cq method, normalized using *Polr2a* as a reference housekeeping gene.

### 2.7. Tissue Microarray Analysis of ERRα and SPP1 Protein Expression

TMA slides including normal human breast tissues and carcinoma tissues were purchased from Novus Biologicals (NBP2-78114; Centennial, CO, USA). These slides were processed for immunohistochemistry using anti-SPP1 and anti-ERRα antibodies as described above. Once prepared, the slides were scanned using an Aperio GT450 v1.3 whole slide scanner (Leica Biosystems, Dear Park, IL, USA) at a 40X magnification and high-quality digital images were used to identify individual tissue cores on the TMA slides using the Qpath 0.4.3 software [43].

### 2.8. Co-Expression Analysis of Esr1, Esrra, and Spp1 Genes in Human Breast Cancer

The co-expression of the *Esr1*, *Esrra*, and *Spp1* genes in human breast cancer was investigated using RNA sequencing data from The Cancer Genome Atlas (TCGA). Specifically, Fragments Per Kilobase of transcript per Million mapped reads (FPKM) values were utilized to assess gene expression levels across a total of 1075 human breast cancer samples. The FPKM data for each gene were obtained from the Human Protein Atlas (https://www.proteinatlas.org, accessed on 2 December 2023). First, we calculated the average FPKM for each gene across all samples and classified samples based on the expression threshold, i.e., samples exhibiting FPKM values less than 25% of the calculated average were categorized as negative for that gene, while those with FPKM values exceeding 25% of the average were classified as positive. Subsequently, co-expression analyses were performed for each gene pair, specifically *Esr1–Esrra*, *Esr1–Spp1*, and *Esrra–Spp1*. The percentage of positive and negative tumors for each gene combination was computed. These results were expressed as a percentage of the total number of tumors analyzed, facilitating a comprehensive understanding of the relationships between gene expressions in breast cancer samples. Data were presented as proportions of positive and negative classifications to allow for straightforward interpretation of gene co-expression patterns within the context of human breast cancer.

### 2.9. Statistical Analysis

Statistical analysis was conducted using GraphPad Prism software v6.0a for Mac (La Jolla, CA, USA). Non-parametric analysis was used to determine equality of variance for the quantitative analysis of tumor number. The incidence of mammary tumors between the control group and the group exposed to GCRsim was compared using a relative risk analysis. A two-tailed *p*-value of less than 0.05 was considered statistically significant. The confidence interval (CI) for the relative risk was calculated to estimate the precision of the effect size. Moreover, the IHC and qPCR data from control and irradiated mice were evaluated for statistical significance using a two-tailed paired student *t*-test. Results are presented as mean ± standard error of the mean (SEM), with a *p*-value < 0.05 considered statistically significant.

## 3. Results

### 3.1. Increased Ductal Overgrowth and Mammary Tumorigenesis After GCRsim

We analyzed the established histological and molecular markers of mammary preneoplastic lesions in normal-appearing mammary gland samples from both sham-treated and GCRsim-exposed *Apc*^Min/+^ mice (Figure 1A). To investigate GCRsim-induced changes in the mammary tissue, we performed whole-mount staining and H&E staining. Whole-mount analysis of mammary gland ductal morphology revealed increased ductal outgrowth in GCRsim-exposed *Apc*^Min/+^ mice compared to the control group (Figure 1B). In line with this observation, histological analysis of H&E-stained mammary tissue showed a significantly higher number of ducts per microscopic field in GCRsim-exposed *Apc*^Min/+^ mice compared to controls (Figure 1B,C). Additionally, the incidence of mammary tumors significantly increased in GCRsim-exposed mice (Figure 1D,E). In the control group (*n* = 40), the incidence of mammary tumors was 5%, while in the GCRsim group (*n* = 25), the incidence increased to 24%. The relative risk of developing mammary tumors in the GCRsim group compared to the control group was 4.8, with a 95% confidence interval (CI) ranging from 1.05 to 21.95, indicating a statistically significant increase in mammary tumor development risk (*p* = 0.043). This suggests that exposure to GCRsim is associated with increased ductal cell proliferation accompanied with a significantly higher risk of mammary tumorigenesis in *Apc*^Min/+^ mice.

### 3.2. GCRsim-Exposure Induces Activation of ERα and Downstream Target Genes

**Figure 2:**
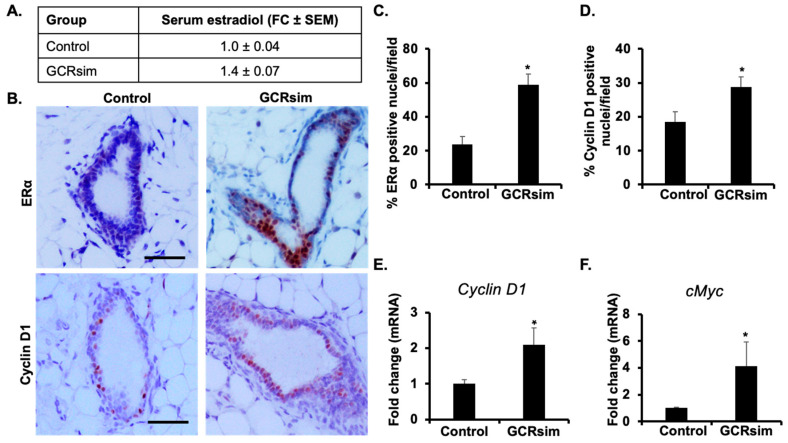
Chronic GCRsim exposure increases serum estradiol levels and enhances estrogen receptor signaling and proliferation markers in mammary tissue. (**A**) Serum estradiol levels expressed as fold change (FC) relative to control animals, demonstrating increased estradiol in GCRsim-exposed mice. (**B**) Representative photomicrographs illustrating increased immunohistochemical staining for estrogen receptor alpha (ERα) and Cyclin D1 in mammary tissues of GCRsim-exposed animals (scale bar = 50 µm). (**C**) Quantification of ERα-positive nuclei in mammary tissue, showing increased receptor expression in the GCRsim group. (**D**) Quantification of Cyclin D1-positive nuclei, indicating increased cellular proliferation in response to GCRsim exposure. (**E**) Fold change in mRNA expression of ERα downstream target gene Cyclin D1 (or *Ccnd1*), demonstrating activation of estrogen signaling. (**F**) Fold change in mRNA expression of ERα downstream target gene *cMyc*, indicating enhanced proliferative signaling. Statistically significant difference (*p* < 0.05) relative to the control group is indicated by an asterisk (*).

To determine whether GCRsim-exposure can alter estrogen and associated ERα signaling, we first analyzed serum estradiol concentrations in the GCRsim irradiated mice at 100–110 days post radiation, which was ~1.4-fold higher than in the unirradiated control groups (Figure 2A). Concurrent to systemic increase in estradiol, increased protein expression accompanied with enhanced nuclear localization of ERα and Cyclin D1 in mammary tissues was also noted using immunohistochemical staining in the GCRsim group at 100–110 days post radiation (Figure 2B). Quantification of the immunohistochemical results exhibited significantly increased ERα (Figure 2C) and Cyclin D1 (Figure 2D) positive nuclei in GCRsim-exposed mice in comparison with controls, suggesting GCRsim-induced activation of ERα signaling. Further, we looked into the mRNA expression level of ERα downstream genes, i.e., *Ccnd1* and *cMyc* in mammary tissues, and found a significantly higher expression of *Ccnd1* (Figure 2E) and *cMyc* (Figure 2F) in the GCRsim-irradiated group compared to the respective controls. These results indicate that GCRsim exposure leads to a systemic increase in estradiol levels and activation of ERα signaling in mammary tissues, potentially driving the expression of oncogenic targets such as Cyclin D1 and cMyc.

### 3.3. GCRsim-Exposure Induces Activation of ERRα and Downstream Target Genes

**Figure 3:**
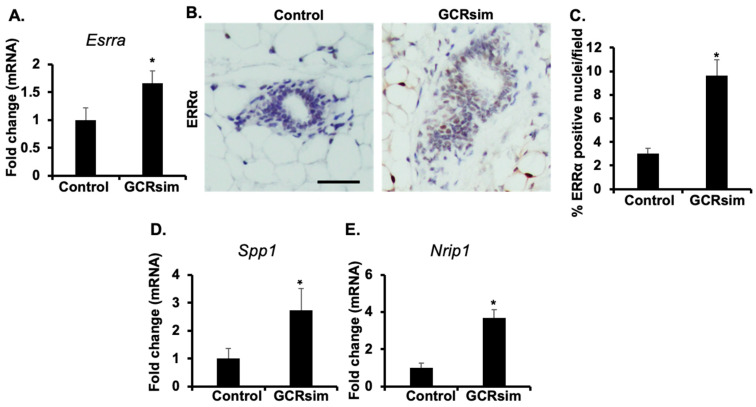
Chronic GCRsim exposure elevates mRNA and protein levels of estrogen-related receptor alpha (ERRα). (**A**) Fold change in mRNA expression of ERRα (or *Esrra*) in mammary tissue of control and GCRsim-exposed mice, showing elevated expression in response to GCRsim exposure. (**B**) Representative photomicrographs of ERRα protein expression in the mammary gland, demonstrating increased levels in GCRsim-exposed mice (scale bar = 50 µm). (**C**) Quantification of ERRα-positive cells per high-power microscopic field (HPF), indicating increased receptor expression in GCRsim-exposed animals. (**D**) Gene expression analysis showing activation of ERRα downstream target *Spp1*, expressed as fold change relative to control. (**E**) Gene expression analysis showing activation of ERRα downstream target Nrip1, expressed as fold change relative to control. Statistically significant change (*p* < 0.05) relative to the control group is denoted by an asterisk (*).

**Figure 4:**
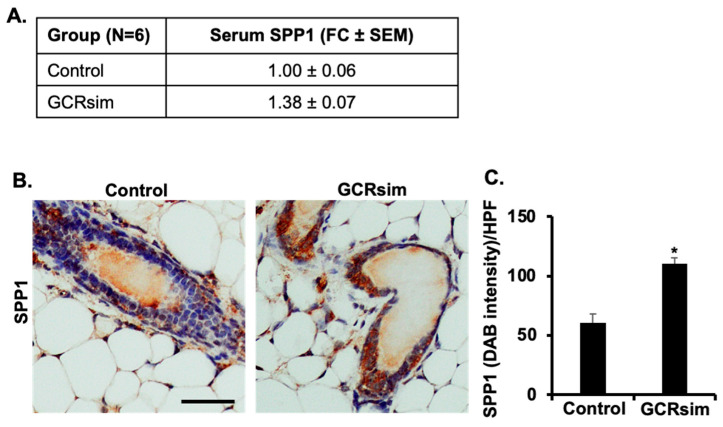
GCRsim exposure elevates SPP1 expression at both systemic and tissue levels. (**A**) Serum levels of SPP1 expressed as fold change relative to the control group, indicating increased levels in GCRsim-exposed mice. (**B**) Representative photomicrographs of mammary gland tissue showing increased SPP1 expression in GCRsim-exposed animals compared to controls (scale bar = 50 µm). (**C**) Quantification of SPP1 signal intensity in mammary tissue, demonstrating enhanced expression in response to GCRsim exposure. Statistically significant change (*p* < 0.05) relative to the control group is denoted by an asterisk (*).

Higher estrogen level is known to activate ERRα directly as well as through ERα-mediated transcriptional activation of *Esrra* gene [38,44,45,46,47,48,49]. In order to relate ERRα with activation of GCR-induced estrogen signaling, we noted a significant increase in mRNA levels of *Esrra* in the GCRsim-irradiated group compared to the controls (Figure 3A). Further, immunohistochemical analysis of ERRα in the mammary tissues of GCRsim-irradiated mice also showed increased nuclear staining, relative to the control group (Figure 3B,C). Additionally, mRNA expression of ERRα downstream transcriptional targets, i.e., *Spp1* (Figure 3D) and *Nrip1* (Figure 3E), was also significantly increased in the GCRsim-irradiated group compared to the control group mice. Next, we looked into the SPP1 protein expression in the serum, and we found a significantly increased level of SPP1 approximately 1.38-fold higher than in the unirradiated control groups (Figure 4A). In addition to overexpression of SPP1 in serum, protein expression of SPP1 was also enhanced in GCRsim-irradiated mice mammary ductal epithelial cells (Figure 4B,C). These findings suggest that GCRsim irradiation enhances ERRα activity and its downstream signaling in mammary tissues, potentially contributing to radiation-induced tumorigenesis through estrogen-related pathways.

### 3.4. ERα, ERRα and SPP1 Protein Expression in Mouse and Human Mammary Tumors

**Figure 5:**
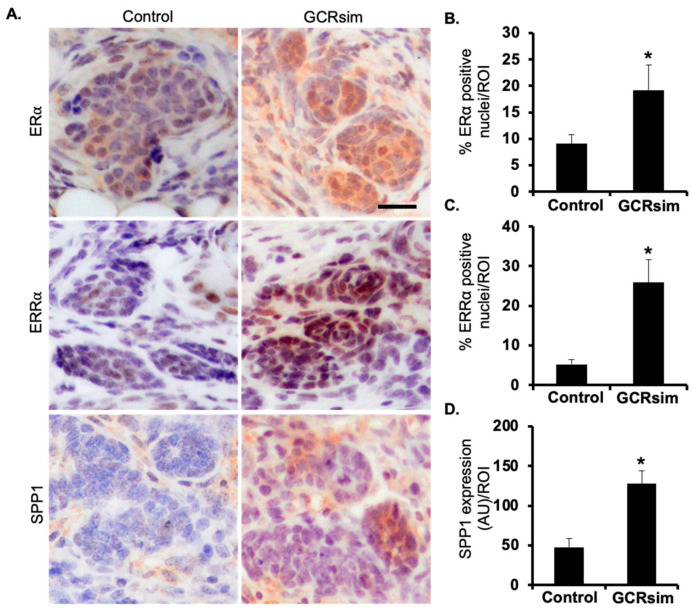
Increased ERα, ERRα, and SPP1 protein expression in GCRsim-induced tumors from *Apc*^Min/+^ mice compared to controls. (**A**) Representative immunohistochemically (IHC) stained images of mammary tissue showing the expression of ERα, ERRα, and SPP1 in both control and GCRsim-induced tumor samples (scale bar = 50 µm). (**B**) Increased tissue expression of ERα in GCRsim-exposed *Apc*^Min/+^ tumors compared to controls. (**C**) Elevated ERRα protein expression in GCRsim-induced tumors compared to controls. (**D**) Enhanced SPP1 expression in GCRsim-exposed tumors relative to control tumors. Statistically significant changes (*p* < 0.05) are denoted by an asterisk (*). AU: Arbitrary unit; ROI: Region of interest.

**Figure 6:**
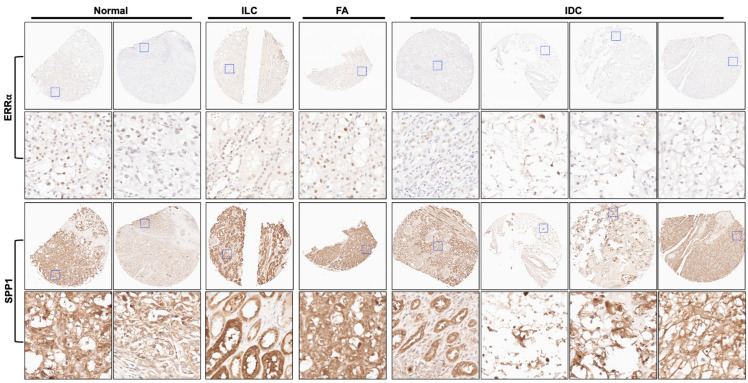
Tissue Microarray (TMA) analysis of ERRα and SPP1 protein expression in normal and malignant human breast tissues. Expression of ERRα and SPP1 in different types of breast tissue, including normal tissue, invasive lobular carcinoma (ILC), fibroadenoma (FA), and invasive ductal carcinoma (IDC). **Upper panels** show representative images of tissue microarray (TMA) cores captured using an Aperio whole-slide digital scanner while **lower panels** show magnified views (marked by blue color box in the respective upper panel) of selected regions with increased expression (scale bar = 100 µm). Comparisons are provided for normal breast tissue and various tumor types to demonstrate a correlation in ERRα and SPP1 expressions.

We investigated the expression pattern of ERα, ERRα and SPP1 in the tumor tissues of both GCRsim exposed and control mice. Notably, a similar pattern in the expression of ERα, ERRα and SPP1 was observed, as they were all overexpressed in the GCRsim-exposed tumors compared to the tumors from control mice (Figure 5A–D). Further, we attempted to ascertain the expression of these protein markers in human breast cancer tissues to test the relevance of these GCRsim-induced proteins in human breast cancer. Interestingly, using serial normal and tumor tissue sections, we found that both ERRα and SPP1 concurrently overexpressed in the different breast cancer tissues including invasive lobular carcinoma, fibroadenoma, and invasive ductal carcinoma compared to the normal breast tissues (Figure 6). Notably, ERRα was localized to the nucleus and SPP1 expression was generally cytoplasmic with a higher likelihood of co-expression in tumor samples than in the normal breast tissue. These findings suggest that the overexpression of ERRα and SPP1 in GCRsim-induced tumors mirrors their elevated expression in human breast cancers, highlighting their potential relevance as biomarkers or therapeutic targets in radiation-induced and spontaneous breast tumorigenesis.

### 3.5. Co-Expression of Spp1 in ERα and ERRα Positive Human Breast Cancer

**Figure 7:**
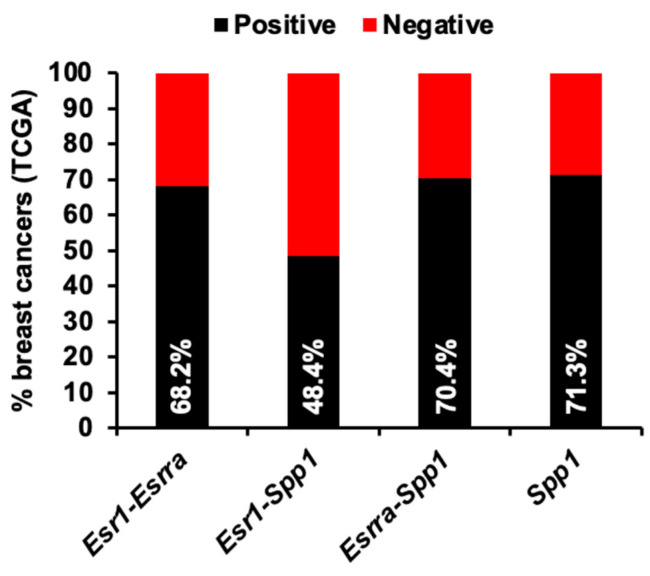
Co-expression analysis of *Esr1*, *Esrra*, and *Spp1* genes in human breast cancer using RNA-Seq data from The Cancer Genome Atlas (TCGA). Presented bar graph illustrates the co-expression patterns of the genes *Esr1*, *Esrra*, and *Spp1* in human breast cancer samples, analyzed using RNA sequencing (RNA-Seq) data from TCGA. Samples were categorized based on Fragments Per Kilobase of transcript per Million mapped reads (FPKM) values as negative (i.e., samples with FPKM values in the lowest quartile, specifically *Esr1* FPKM ≤ 10.1, *Esrra* FPKM ≤ 2.45, and *Spp1* FPKM ≤ 30.22) and positive (i.e., samples with FPKM values above the 25th percentile cutoff). This analysis provides insight into how these genes are co-expressed and their relationship in the context of human breast cancer progression.

The protein ERα is a known transcriptional regulator of *Esrra* and *Spp1* genes [36,37,38,39,40]. Additionally, ERRα is also a transcriptional regulator of the *Spp1* gene [38,44,45,46,47,48,49] (Table S2). To understand the crosstalk between expression pattens of Spp1 upstream factors, i.e., ERα and ERRα in breast cancer, analysis of RNAseq data from a total of 1075 human breast cancer samples revealed a higher co-expression of *Esrra* and *Spp1* (70.4% of total tumors) compared to relatively lower co-expression of *Esr1* and *Spp1* (48.4% of total tumors). This indicates that upregulation/activation of both ERα and ERRα is important for SPP1 positive breast cancer cells (Figure 7). These results suggest that the co-activation of both ERα and ERRα plays a crucial role in regulating SPP1 expression in breast cancer, with ERRα potentially being a more dominant regulator in SPP1-positive tumors in both mouse and human mammary tumors (Figure 5, Figure 6 and Figure 7).

## 4. Discussion

**Figure 8:**
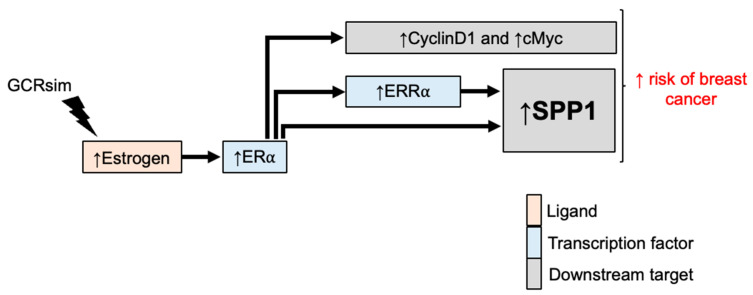
Schematic representation of putative molecular pathways linking GCRsim-induced estrogen response to increased breast cancer risk. GCRsim exposure results in: elevated estrogen levels, enhancing estrogen signaling in breast tissues involving activation of ERα, ERRα, and proliferation markers such as Cyclin D1 and cMyc. Both ERα and ERRα could induce overexpression of SPP1 at both systemic and tissue levels, ultimately contributing to enhanced breast cancer risk. Arrows in the schematic indicate the putative molecular connections between GCRsim-induced changes, receptor upregulation, proliferative signaling, and tumor development.

Our findings demonstrate increased ductal outgrowth and duct density, along with a higher incidence of mammary tumors following GCRsim exposure. Further investigation into estrogenic responses revealed that GCRsim exposure led to a sustained elevation in serum estradiol levels, coupled with enhanced activation of ERα and its downstream targets, which are known to promote cell proliferation in mammary ductal epithelial cells. In parallel with increased ERα activation, we observed significant overexpression and nuclear localization of ERRα in GCRsim-exposed mouse mammary tissues. Moreover, elevated expression of its downstream target, *Spp1* gene and protein, supports the involvement of ERRα in GCRsim-induced mammary tumorigenesis. Notably, the concurrent upregulation of ERα, ERRα, and SPP1 suggests a crosstalk between estrogen signaling and inflammatory pathways mediated by SPP1 in the context of GCRsim exposure [28,31,38,47,48,49]. Collectively, these results suggest that GCRsim exposure induces a sustained estrogenic response, promoting both preneoplastic and neoplastic changes involving ERα, ERRα, and SPP1 signaling axis (Figure 8).

A high level of bioavailable estradiol is a risk factor for breast cancer development [50,51]. It has been reported that sublethal IR exposure enhances the aberrant proliferation of ductal epithelial cells, and risk of ER-positive mammary tumor development by activating PER signaling in both animal models and atomic bomb survivors [19,50,52,53]. Our results show a significant increase in serum estradiol concentrations and ERα protein expression, and nuclear localization in GCRsim-irradiated mice compared to controls. The concurrent increase in downstream target genes, like *Ccnd1* and *cMyc*, further underscores the activation of ERα signaling. This observation is consistent with prior findings that radiation exposure enhances ER-positive tumor formation [19]. These findings align with the role of estrogen signaling in breast cancer progression by activating genes such as *Ccnd1* and *cMyc* [54,55,56].

The DNA-binding domains of ERRα and ERα display ~70% homology but only 36% similarity in ligand-binding domain, and therefore, direct activation of ERRα by estradiol is not expected, and estrogen response is likely to be primarily through ERα activation [57,58]. Notably, using chromatin immunoprecipitation assay, the interaction between ERα and multi-hormone response elements (MHREs) present in the ERRα gene promoter region has been demonstrated earlier, and estrogen is also known to augment the association of ERα and MHREs in vivo [44]. Therefore, binding of ERα on ERRα MHRE in response to higher estradiol bioavailability is likely to co-upregulate both ERα and ERRα in response to GCRsim exposure. ERRα is known to regulate SPP1 expression via a non-canonical ERRα response element, and this regulation is dependent on the specific cellular context [39]. SPP1 overexpression has been implicated in mammary cancer development [27], and our study corroborates these findings by showing enhanced SPP1 protein levels in both serum and mammary ductal epithelial cells of GCRsim-exposed mice. Interestingly, SPP1 has been associated with tumor burden and reduced survival in breast cancer patients [59].

In concurrence to activation of ERα, ERRα, and SPP1 signaling axis in GCRsim-induced tumors, using TMA, we noted elevated expression of ERRα and SPP1 in human breast cancer tissues, including invasive lobular carcinoma and invasive ductal carcinoma. This co-expression of SPP1 and ERRα, particularly in ERα and ERRα-positive breast cancer cells, suggests that upregulation of ERRα is critical for SPP1-positive preneoplastic and neoplastic cells [27,60]. Furthermore, the higher co-expression of *Esr1*, *Esrra* and *Spp1* mRNA in human breast cancers emphasizes the importance of ERRα in conjunction with ERα in driving SPP1 expression and, potentially, breast cancer progression. Notably, ERRα inhibitors have also shown potential in breast cancer risk prevention and mitigation through disruption of cancer cell metabolism, proliferation, and tumor growth by interfering with genes associated with oncogenic processes [39,61,62]. Therefore, further studies are required to test and validate the efficacy of ERRα inhibitors in breast cancer prevention after GCRsim exposure to safeguard female astronauts.

Overall, our findings provide novel insights into the effects of GCRsim on mammary gland biology, emphasizing the role of estrogenic and inflammatory signaling in mediating GCRsim-induced preneoplastic and neoplastic changes. The increased ductal outgrowth and tumor incidence observed in the *Apc*^Min/+^ mice suggest that GCRsim exposure leads to alterations in mammary gland architecture and microenvironment, promoting neoplastic transformation. The persistent estrogenic response, activation of ERα and ERRα, and overexpression of SPP1 following GCRsim exposure indicate a complex interplay between hormonal signaling, inflammation, and cancer development. Future studies should aim to dissect the exact molecular mechanisms linking radiation exposure to hormonal dysregulation and inflammatory responses. Additionally, it would be valuable to evaluate the potential of pharmacological interventions, such as selective estrogen receptor modulators (SERMs), estrogen receptor degraders, and aromatase inhibitors, to mitigate estrogen-related mammary tumorigenesis after GCRsim exposure [63,64]. These insights have important implications for understanding breast cancer risk in individuals exposed to space radiation and may help in developing strategies to mitigate these risks.

## 5. Conclusions

In conclusion, our study demonstrates that GCRsim exposure induces ductal overgrowth, increased duct density, and a heightened incidence of mammary tumors in *Apc*^Min/+^ mice. Chronic increase in estradiol levels and the concurrent activation of ERα–ERRα signaling in GCRsim-irradiated mice highlight the influence of estrogenic responses in promoting mammary ductal epithelial cell proliferation. Additionally, ERRα expression, particularly through its regulatory effects on SPP1, underscores the potential importance of estrogen and inflammatory crosstalk in IR-induced breast cancer. Our analysis of human breast cancer samples revealed elevated expression of ERRα and SPP1, mirroring the findings in GCRsim-exposed mammary tissues and suggesting the relevance of these markers in both IR-induced and spontaneous breast cancers. The co-expression of ERα and ERRα, alongside SPP1 upregulation, points to a complex interplay of hormonal and inflammatory signaling that could underlie mammary carcinogenesis following radiation exposure. Overall, this study provides insights into the effects of GCRsim on mammary tissue biology, with implications for breast cancer risk after space radiation. Future research should focus on the detailed mechanisms linking radiation to hormonal dysregulation and inflammatory responses and evaluate the efficacy of pharmacological interventions, such as anti-estrogens or ERRα inhibitors, in mitigating GCRsim-induced carcinogenesis. These findings could contribute to protective strategies for astronauts and others at risk of radiation exposure, ultimately helping to reduce breast cancer incidence.

## Data Availability

All relevant data have been included in this manuscript and supplementary information.

## Supplementary Materials

The following supporting information can be downloaded at: https://www.mdpi.com/article/10.3390/cancers16233954/s1. Table S1: ERα and ERRα downstream target genes identified using TFLink database; and Table S2: Sequence details of qPCR primers.

## References

[1] Barr Y.R., Bacal K., Jones J.A., Hamilton D.R. (2007). Breast cancer and spaceflight: Risk and management. Aviat. Space Environ. Med..

[2] Cucinotta F.A. (2024). Non-targeted effects and space radiation risks for astronauts on multiple International Space Station and lunar missions. Life Sci. Space Res..

[3] Gu Y., Wang J., Wang Y., Xu C., Liu Y., Du L., Wang Q., Ji K., He N., Zhang M. (2023). Association of low-dose ionising radiation with site-specific solid cancers: Chinese medical X-ray workers cohort study, 1950–1995. Occup. Environ. Med..

[4] Little M.P., Hamada N. (2022). Low-Dose Extrapolation Factors Implied by Mortality and Incidence Data from the Japanese Atomic Bomb Survivor Life Span Study Data. Radiat. Res..

[5] Walsh L., Hafner L., Straube U., Ulanowski A., Fogtman A., Durante M., Weerts G., Schneider U. (2021). A bespoke health risk assessment methodology for the radiation protection of astronauts. Radiat. Environ. Biophys..

[6] Kaiser J.C., Jacob P., Meckbach R., Cullings H.M. (2012). Breast cancer risk in atomic bomb survivors from multi-model inference with incidence data 1958–1998. Radiat. Environ. Biophys..

[7] Ramos R.L., Carante M.P., Ferrari A., Sala P., Vercesi V., Ballarini F. (2023). A Mission to Mars: Prediction of GCR Doses and Comparison with Astronaut Dose Limits. Int. J. Mol. Sci..

[8] Ballarini F., Battistoni G., Cerutti F., Fassò A., Ferrari A., Gadioli E., Garzelli M.V., Mairani A., Ottolenghi A., Paretzke H. (2006). GCR and SPE organ doses in deep space with different shielding: Monte Carlo simulations based on the FLUKA code coupled to anthropomorphic phantoms. Adv. Space Res..

[9] Ramos R.L., Carante M.P., Bernardini E., Ferrari A., Sala P., Vercesi V., Ballarini F. (2024). A method to predict space radiation biological effectiveness for non-cancer effects following intense Solar Particle Events. Life Sci. Space Res..

[10] Huff J.L., Poignant F., Rahmanian S., Khan N., Blakely E.A., Britten R.A., Chang P., Fornace A.J., Hada M., Kronenberg A. (2023). Galactic cosmic ray simulation at the NASA space radiation laboratory-Progress, challenges and recommendations on mixed-field effects. Life Sci. Space Res..

[11] Simonsen L.C., Slaba T.C., Guida P., Rusek A. (2020). NASA’s first ground-based Galactic Cosmic Ray Simulator: Enabling a new era in space radiobiology research. PLoS Biol..

[12] Naito M., Kodaira S., Ogawara R., Tobita K., Someya Y., Kusumoto T., Kusano H., Kitamura H., Koike M., Uchihori Y. (2020). Investigation of shielding material properties for effective space radiation protection. Life Sci. Space Res..

[13] Kumar K., Fornace A.J., Suman S. (2024). 8-OxodG: A Potential Biomarker for Chronic Oxidative Stress Induced by High-LET Radiation. DNA.

[14] Suman S., Jaruga P., Dizdaroglu M., Fornace A.J., Datta K. (2020). Heavy ion space radiation triggers ongoing DNA base damage by downregulating DNA repair pathways. Life Sci. Space Res..

[15] Kumar K., Datta K., Fornace A.J., Suman S. (2022). Total body proton and heavy-ion irradiation causes cellular senescence and promotes pro-osteoclastogenic activity in mouse bone marrow. Heliyon.

[16] Suman S., Kumar S., Moon B.H., Angdisen J., Kallakury B.V.S., Datta K., Fornace A.J. (2021). Effects of dietary aspirin on high-LET radiation-induced prostaglandin E2 levels and gastrointestinal tumorigenesis in Apc1638N/+ mice. Life Sci. Space Res..

[17] Rahmanian S., Slaba T.C. (2023). Applicability of the NASA galactic cosmic ray simulator for mice, rats, and minipigs. Acta Astronaut..

[18] Imaoka T., Okamoto M., Nishimura M., Nishimura Y., Ootawara M., Kakinuma S., Tokairin Y., Shimada Y. (2006). Mammary tumorigenesis in ApcMin/+ mice is enhanced by X irradiation with a characteristic age dependence. Radiat. Res..

[19] Suman S., Shuryak I., Kallakury B., Brenner D.J., Fornace A.J., Johnson M.D., Datta K. (2020). Protons Show Greater Relative Biological Effectiveness for Mammary Tumorigenesis with Higher ERα- and HER2-Positive Tumors Relative to γ-rays in APCMin/+ Mice. Int. J. Radiat. Oncol. Biol. Phys..

[20] Kumar K., Moon B.H., Datta K., Fornace A.J., Suman S. (2023). Simulated galactic cosmic radiation (GCR)-induced expression of Spp1 coincide with mammary ductal cell proliferation and preneoplastic changes in ApcMin/+ mouse. Life Sci. Space Res..

[21] Miziak P., Baran M., Błaszczak E., Przybyszewska-Podstawka A., Kałafut J., Smok-Kalwat J., Dmoszyńska-Graniczka M., Kiełbus M., Stepulak A. (2023). Estrogen Receptor Signaling in Breast Cancer. Cancers.

[22] Mangani S., Piperigkou Z., Koletsis N.E., Ioannou P., Karamanos N.K. (2024). Estrogen receptors and extracellular matrix: The critical interplay in cancer development and progression. FEBS J..

[23] Lin C.Y., Ström A., Vega V.B., Kong S.L., Yeo A.L., Thomsen J.S., Chan W.C., Doray B., Bangarusamy D.K., Ramasamy A. (2004). Discovery of estrogen receptor alpha target genes and response elements in breast tumor cells. Genome Biol..

[24] Datta K., Hyduke D.R., Suman S., Moon B.H., Johnson M.D., Fornace A.J. (2012). Exposure to ionizing radiation induced persistent gene expression changes in mouse mammary gland. Radiat. Oncol..

[25] Suman S., Johnson M.D., Fornace A.J., Datta K. (2012). Exposure to ionizing radiation causes long-term increase in serum estradiol and activation of PI3K-Akt signaling pathway in mouse mammary gland. Int. J. Radiat. Oncol. Biol. Phys..

[26] Hubbard N.E., Chen Q.J., Sickafoose L.K., Wood M.B., Gregg J.P., Abrahamsson N.M., Engelberg J.A., Walls J.E., Borowsky A.D. (2013). Transgenic mammary epithelial osteopontin (spp1) expression induces proliferation and alveologenesis. Genes Cancer.

[27] Göthlin Eremo A., Lagergren K., Othman L., Montgomery S., Andersson G., Tina E. (2020). Evaluation of SPP1/osteopontin expression as predictor of recurrence in tamoxifen treated breast cancer. Sci. Rep..

[28] Wei T., Bi G., Bian Y., Ruan S., Yuan G., Xie H., Zhao M., Shen R., Zhu Y., Wang Q. (2020). The Significance of Secreted Phosphoprotein 1 in Multiple Human Cancers. Front. Mol. Biosci..

[29] Hu P., Kinyamu H.K., Wang L., Martin J., Archer T.K., Teng C. (2008). Estrogen induces estrogen-related receptor alpha gene expression and chromatin structural changes in estrogen receptor (ER)-positive and ER-negative breast cancer cells. J. Biol. Chem..

[30] Stein R.A., Chang C.Y., Kazmin D.A., Way J., Schroeder T., Wergin M., Dewhirst M.W., McDonnell D.P. (2008). Estrogen-related receptor alpha is critical for the growth of estrogen receptor-negative breast cancer. Cancer Res..

[31] Tripathi M., Singh B.K. (2023). Metabolic switching of estrogen-related receptor alpha in breast cancer aggression. FEBS J..

[32] Misawa A., Inoue S. (2015). Estrogen-Related Receptors in Breast Cancer and Prostate Cancer. Front. Endocrinol..

[33] Fradet A., Sorel H., Bouazza L., Goehrig D., Dépalle B., Bellahcène A., Castronovo V., Follet H., Descotes F., Aubin J.E. (2011). Dual function of ERRα in breast cancer and bone metastasis formation: Implication of VEGF and osteoprotegerin. Cancer Res..

[34] Giguère V. (2008). Transcriptional control of energy homeostasis by the estrogen-related receptors. Endocr. Rev..

[35] Vanacker J.M., Pettersson K., Gustafsson J.A., Laudet V. (1999). Transcriptional targets shared by estrogen receptor-related receptors (ERRs) and estrogen receptor (ER) alpha, but not by ERbeta. EMBO J..

[36] Yevshin I., Sharipov R., Kolmykov S., Kondrakhin Y., Kolpakov F. (2019). GTRD: A database on gene transcription regulation-2019 update. Nucleic Acids Res..

[37] Yevshin I., Sharipov R., Valeev T., Kel A., Kolpakov F. (2017). GTRD: A database of transcription factor binding sites identified by ChIP-seq experiments. Nucleic Acids Res..

[38] Vanacker J.M., Delmarre C., Guo X., Laudet V. (1998). Activation of the osteopontin promoter by the orphan nuclear receptor estrogen receptor related alpha. Cell Growth Differ..

[39] Zirngibl R.A., Chan J.S., Aubin J.E. (2008). Estrogen receptor-related receptor alpha (ERRalpha) regulates osteopontin expression through a non-canonical ERRalpha response element in a cell context-dependent manner. J. Mol. Endocrinol..

[40] Liska O., Bohár B., Hidas A., Korcsmáros T., Papp B., Fazekas D., Ari E. (2022). TFLink: An integrated gateway to access transcription factor-target gene interactions for multiple species. Database.

[41] Norbury J.W., Schimmerling W., Slaba T.C., Azzam E.I., Badavi F.F., Baiocco G., Benton E., Bindi V., Blakely E.A., Blattnig S.R. (2016). Galactic cosmic ray simulation at the NASA Space Radiation Laboratory. Life Sci. Space Res..

[42] Crowe A.R., Yue W. (2019). Semi-quantitative Determination of Protein Expression using Immunohistochemistry Staining and Analysis: An Integrated Protocol. Bio Protoc..

[43] Bankhead P., Loughrey M.B., Fernández J.A., Dombrowski Y., McArt D.G., Dunne P.D., McQuaid S., Gray R.T., Murray L.J., Coleman H.G. (2017). QuPath: Open source software for digital pathology image analysis. Sci. Rep..

[44] Liu D., Zhang Z., Gladwell W., Teng C.T. (2003). Estrogen stimulates estrogen-related receptor alpha gene expression through conserved hormone response elements. Endocrinology.

[45] Cvoro A., Paruthiyil S., Jones J.O., Tzagarakis-Foster C., Clegg N.J., Tatomer D., Medina R.T., Tagliaferri M., Schaufele F., Scanlan T.S. (2007). Selective activation of estrogen receptor-beta transcriptional pathways by an herbal extract. Endocrinology.

[46] Han H., Cho J.W., Lee C., Yun A., Kim H., Bae D., Yang S., Kim C.Y., Lee M., Kim E. (2018). TRRUST v2: An expanded reference database of human and mouse transcriptional regulatory interactions. Nucleic Acids Res..

[47] Nichol D., Christian M., Steel J.H., White R., Parker M.G. (2006). RIP140 expression is stimulated by estrogen-related receptor alpha during adipogenesis. J. Biol. Chem..

[48] Park E., Gong E.Y., Romanelli M.G., Lee K. (2012). Suppression of estrogen receptor-alpha transactivation by thyroid transcription factor-2 in breast cancer cells. Biochem. Biophys. Res. Commun..

[49] Rajalin A.M., Pollock H., Aarnisalo P. (2010). ERRalpha regulates osteoblastic and adipogenic differentiation of mouse bone marrow mesenchymal stem cells. Biochem. Biophys. Res. Commun..

[50] Kabuto M., Akiba S., Stevens R.G., Neriishi K., Land C.E. (2000). A prospective study of estradiol and breast cancer in Japanese women. Cancer Epidemiol. Biomark. Prev..

[51] Key T.J. (1999). Serum oestradiol and breast cancer risk. Endocr. Relat. Cancer.

[52] Grant E.J., Cologne J.B., Sharp G.B., Eguchi H., Stevens R.G., Izumi S., Kim Y.M., Berrington de González A., Ohishi W., Nakachi K. (2018). Bioavailable serum estradiol may alter radiation risk of postmenopausal breast cancer: A nested case-control study. Int. J. Radiat. Biol..

[53] Grant E.J., Neriishi K., Cologne J., Eguchi H., Hayashi T., Geyer S., Izumi S., Nishi N., Land C., Stevens R.G. (2011). Associations of ionizing radiation and breast cancer-related serum hormone and growth factor levels in cancer-free female A-bomb survivors. Radiat. Res..

[54] Wang C., Mayer J.A., Mazumdar A., Fertuck K., Kim H., Brown M., Brown P.H. (2011). Estrogen induces c-myc gene expression via an upstream enhancer activated by the estrogen receptor and the AP-1 transcription factor. Mol. Endocrinol..

[55] Casimiro M.C., Wang C., Li Z., Di Sante G., Willmart N.E., Addya S., Chen L., Liu Y., Lisanti M.P., Pestell R.G. (2013). Cyclin D1 determines estrogen signaling in the mammary gland in vivo. Mol. Endocrinol..

[56] Kilker R.L., Hartl M.W., Rutherford T.M., Planas-Silva M.D. (2004). Cyclin D1 expression is dependent on estrogen receptor function in tamoxifen-resistant breast cancer cells. J. Steroid. Biochem. Mol. Biol..

[57] Wei W., Schwaid A.G., Wang X., Wang X., Chen S., Chu Q., Saghatelian A., Wan Y. (2016). Ligand Activation of ERRα by Cholesterol Mediates Statin and Bisphosphonate Effects. Cell. Metab..

[58] Giguère V. (2002). To ERR in the estrogen pathway. Trends Endocrinol. Metab..

[59] Singhal H., Bautista D.S., Tonkin K.S., O’Malley F.P., Tuck A.B., Chambers A.F., Harris J.F. (1997). Elevated plasma osteopontin in metastatic breast cancer associated with increased tumor burden and decreased survival. Clin. Cancer Res..

[60] Castello L.M., Raineri D., Salmi L., Clemente N., Vaschetto R., Quaglia M., Garzaro M., Gentilli S., Navalesi P., Cantaluppi V. (2017). Osteopontin at the Crossroads of Inflammation and Tumor Progression. Mediators. Inflamm..

[61] Chang C.Y., Kazmin D., Jasper J.S., Kunder R., Zuercher W.J., McDonnell D.P. (2011). The metabolic regulator ERRα, a downstream target of HER2/IGF-1R, as a therapeutic target in breast cancer. Cancer Cell.

[62] Park S., Safi R., Liu X., Baldi R., Liu W., Liu J., Locasale J.W., Chang C.Y., McDonnell D.P. (2019). Inhibition of ERRα Prevents Mitochondrial Pyruvate Uptake Exposing NADPH-Generating Pathways as Targetable Vulnerabilities in Breast Cancer. Cell Rep..

[63] Ascione L., Castellano G., Curigliano G., Zagami P. (2024). Endocrine therapy for early breast cancer in the era of oral selective estrogen receptor degraders: Challenges and future perspectives. Curr. Opin. Oncol..

[64] Guglielmi G., Re M.D., Gol L.S., Bengala C., Danesi R., Fogli S. (2024). Pharmacological insights on novel oral selective estrogen receptor degraders in breast cancer. Eur. J. Pharmacol..

