# Supplementary material for: Simulated Galactic Cosmic Radiation Exposure-Induced Mammary Tumorigenesis in ApcMin/+ Mice Coincides with Activation of ERα-ERRα-SPP1 Signaling Axis
Source: Cancers (Basel). 2024 Nov 26;16(23):3954. doi: 10.3390/cancers16233954 (PMC11640058; doi:10.3390/cancers16233954)
Supplement: Supplementary file 1 [file cancers-16-03954-s001.zip › cancers-3327227-supplementary.pdf]

Table S1: Sequence details of qPCR primers

Gene symbol	Assay Name*	Primer Sequence
Esr1	Mm.PT.58.8025728	5’-TGT GTG CCT CAA ATC CAT CA-3’<br>5’-AGT GTC TGT GAT CTT GTC CAG-3’
Esrra	Mm.PT.58.29487425	5’-GAG ACA CCG AAT GCA GAA GA-3’<br>5’-GTG CTC TCC TCT TTC AAG GTC-3’
Nrip1	Mm.PT.58.41249580	5’-CTT CCT TTC CCA CAT AGC AGA-3’<br>5’-CTC GCA ACT TCC TTA GCA CA-3’
cMyc	Mm.PT.58.28494642	5’-TTC TCT CCT TCC TCG GAC TC-3’<br>5’-CTT CCT CAT CTT CTT GCT CTT CT-3’
Ccnd1	Mm.PT.58.28503828	5’-CAA CAA CTT CCT CTC CTG CTA C-3’<br>5’-GCT TCA ATC TGT TCC TGG CA-3’
Polr2a	Mm.PT.39a.22214849	5’-GGT CCT TCG AAT CCG CAT C-3’<br>5’-CAG GGT CAT ATC TGT CAG CAT G-3’

* PrimeTime™ qPCR Assays from Integrated DNA technology (https://www.idtdna.com/)

Table S2: ERα and ERRα downstream target genes identified using *TFLink* database.

Transcription factor (TF)	Target genes*	References
**ERα**	*Esrra*	(Liu et al., 2003; Han et al., 2018; Yevshin et al., 2017)
	Spp1	(Yevshin et al., 2017)
	Ccnd1	(Park et al., 2012; Cvoro et al., 2007; Han et al., 2018; Yevshin et al., 2017)
	cMyc	(Cvoro et al., 2007; Han et al., 2018; Yevshin et al., 2017)
	Nrip1	(Yevshin et al., 2017)
**ERRα**	*Spp1*	(Zirngibl et al., 2008; Rajalin et al., 2010; Han et al., 2018; Vanacker et al., 1998)
	Nrip1	(Nichol et al., 2006; Han et al., 2018)

*Identified using TFLink (https://tflink.net/) database (Liska et al., 2022)

References

1. Cvoro, A. et al. (2007) Selective activation of estrogen receptor-beta transcriptional pathways by an herbal extract. *Endocrinology*, **148**, 538-547.
2. Han, H. et al. (2018) TRRUST v2: an expanded reference database of human and mouse transcriptional regulatory interactions. *Nucleic Acids Res*, **46**, D380-D386.
3. Liska, O. et al. (2022) TFLink: an integrated gateway to access transcription factor-target gene interactions for multiple species. *Database (Oxford)*, **2022**, baac083.
4. Liu, D. et al. (2003) Estrogen stimulates estrogen-related receptor alpha gene expression through conserved hormone response elements. *Endocrinology*, **144**, 4894-4904.
5. Nichol, D. et al. (2006) RIP140 expression is stimulated by estrogen-related receptor alpha during adipogenesis. *J Biol Chem*, **281**, 32140-32147.
6. Park, E. et al. (2012) Suppression of estrogen receptor-alpha transactivation by thyroid transcription factor-2 in breast cancer cells. *Biochem Biophys Res Commun*, **421**, 532-537.
7. Rajalin, A.M., Pollock, H. & Aarnisalo, P. (2010) ERRalpha regulates osteoblastic and adipogenic differentiation of mouse bone marrow mesenchymal stem cells. *Biochem Biophys Res Commun*, **396**, 477-482.
8. Vanacker, J.M. et al. (1998) Activation of the osteopontin promoter by the orphan nuclear receptor estrogen receptor related alpha. *Cell Growth Differ*, **9**, 1007-1014.
9. Yevshin, I. et al. (2017) GTRD: a database of transcription factor binding sites identified by ChIP-seq experiments. *Nucleic Acids Res*, **45**, D61-D67.
10. Zirngibl, R.A., Chan, J.S. & Aubin, J.E. (2008) Estrogen receptor-related receptor alpha (ERRalpha) regulates osteopontin expression through a non-canonical ERRalpha response element in a cell context-dependent manner. *J Mol Endocrinol*, **40**, 61-73.
